# The association between non-viral sexually transmitted infections and pregnancy outcome in Latin America and the Caribbean: A systematic review

**DOI:** 10.1016/j.heliyon.2023.e23338

**Published:** 2023-12-13

**Authors:** Maria Lara-Escandell, Carlotta Gamberini, Naomi C.A. Juliana, Salwan Al-Nasiry, Servaas A. Morré, Elena Ambrosino

**Affiliations:** a Institute for Public Health Genomics (IPHG), Department of Genetics and Cell Biology, Research School GROW for Oncology and Reproduction, Faculty of Health, Medicine & Life Sciences, University of Maastricht, 6229 ER Maastricht, the Netherlands; b Department of Molecular and Cellular Engineering, Jacob Institute of Biotechnology and Bioengineering, Sam Higginbottom University of Agriculture, Technology and Sciences, Allahabad 211007, UP, India; c Department of Obstetrics and Gynecology, Maastricht University Medical Centre+, Maastricht, the Netherlands; d Dutch Chlamydia Trachomatis Reference Laboratory on Behalf of the Epidemiology and Surveillance Unit, Centre for Infectious Disease Control, National Institute for Public Health and the Environment, 3721 MA Bilthoven, the Netherlands

**Keywords:** Sexually transmitted infections (STI), Adverse pregnancy outcomes, Latin America and the Caribbean (LAC)

## Abstract

**Introduction:**

Non-viral sexually transmitted infections are known to be associated with adverse pregnancy outcomes. For these pathogens, standard antenatal screening is not broadly performed in Latin America and the Caribbean. The aim of this study was to comprehensively review the association of non-viral sexually transmitted infections and neonatal outcomes among pregnant women in the region.

**Methods:**

Four databases (PubMed, Embase, SciELO and LILACS) were examined to identify eligible studies published up to September 2022. English or Spanish cross-sectional, case-control and cohort studies assessing the association of non-viral sexually transmitted infections and adverse pregnancy outcomes were evaluated. Articles were firstly screened by means of title and abstract. Potential articles were fully read and assessed for inclusion according to the eligibility criteria. Snowballing search was performed by screening of bibliographies of the chosen potentially relevant papers. Risk of bias within studies was assessed using the Joanna Briggs Institute reviewer's manual.

**Results:**

A selection of 10 out of 9772 search records from five Latin America and the Caribbean countries were included. Six studies associated *Treponema pallidum* infection with preterm birth (1/6), history of previous spontaneous abortion (2/6), fetal and infant death (1/6), low birth weight (1/6) and funisitis of the umbilical cord (1/6). Three studies associated *Chlamydia trachomatis* infection with preterm birth (2/3), ectopic pregnancy (1/3) and respiratory symptoms on the newborn (1/3). One study associated *Mycoplasma genitalium* infection with preterm birth.

**Conclusion:**

This review provides evidence on the association of non-viral sexually transmitted infections with adverse pregnancy outcomes. Further investigation is needed to establish more associations between non-viral sexually transmitted infections and pregnancy outcome, especially for *Mycoplasma genitalium*, *Trichomonas vaginalis* and *Neisseria gonorrhoeae*. Overall, this review calls for more research for public health interventions to promote screening of non-viral sexually transmitted infections during pregnancy, among high-risk population groups of pregnant women living in the region.

## 1 Introduction

Syphilis, gonorrhea, chlamydia and trichomoniasis are non-viral sexually transmitted infections (STIs) which remain the most common STIs, despite infection can be prevented by using barrier contraceptive methods during vaginal, anal or oral sex [[1], [2], [3], [4]]. They are respectively caused by the bacteria *Treponema pallidum*, *Neisseria gonorrhoeae*, *Chlamydia trachomatis* and the protozoan parasite *Trichomonas vaginalis*. *Mycoplasma genitalium* has been considered an emerging STI for the past 10 years although its incidence among the global population is unknown due to lack of testing [5,6]. Among pregnant women, regional estimates of these STIs prevalence vary depending on the global region [4,7]: for syphilis, from 1.1 % (Asia) to 6.5 % (Southern Africa); for chlamydia, from 0.8 % (Asia) to 11.2 % (Latin America); for gonorrhea, from 1.2 % (Latin America) to 4.6 % (Southern Africa); and for trichomoniasis from 3.9 % (Latin America) to 24.6 % (Southern Africa). For *Mycoplasma genitalium* infection, data per world region has still not been reported but regional estimates range from 0.7 % in the United Kingdom, to 5.2 % in Argentina to 11.9 % in the Solomon Islands [5,8].

Although it is difficult to fully elucidate their clinical impact during pregnancy, multiple studies have found associations between these STIs and increased risk of poor maternal and neonatal outcomes (S1 Table) [9]. Untreated syphilis in pregnancy leads to adverse outcomes including early fetal loss, stillbirth, prematurity, low birth weight, neonatal and infant death and congenital disease among newborns [10]. For chlamydia, there is evidence that it may contribute to premature rupture of membranes, preterm labor and birth, low birth weight and stillbirth, as well as conjunctivitis and pneumonitis in infants [11]. Maternal gonorrhea is associated with premature birth, and if perinatal transmission occurs, blindness and joint and bloodstream infections in the newborn [12]. Trichomoniasis has been associated with preterm birth, but it has not been clarified yet whether it causes other complications in pregnancy [13]. The role of *Mycoplasma genitalium* in maternal infections and its impact on pregnancy outcome have only been little evaluated [14]. Existing evidence differs, despite some data suggests it could be associated with spontaneous preterm birth [8,15].

Non-viral STIs are curable and do not influence pregnancy outcomes if treated [16]. Globally, 14 countries recommend screening for chlamydial, gonorrheal and trichomonal infection among pregnant women, being The Bahamas, the only Latin American and Caribbean (LAC) country included [17]. The World Health Organization (WHO) recommends screening for syphilis but has no specific guidelines for the other previously mentioned STIs beyond syndromic management, which advises treatment only to symptomatic women [1]. Syndromic management is the most common approach to treat pregnant women, especially in low- and middle-income countries (LMICs) (S1 Table) [1,7]. Despite this, the frequently asymptomatic nature of STIs is well established, and this approach fails to accurately diagnose most infected women [7,9,[18], [19], [20]].

Control of non-viral STIs in the LAC region, remans challenging [[21], [22], [23]]. For pregnant women, screening for syphilis and human immunodeficiency virus (HIV) is strongly advised during antenatal care, where significant improvements have been made towards the elimination of mother-to-child transmission (MTCT) of these STIs through the years [[24], [25], [26]]. Syphilis screening reached 69 % of coverage in the whole LAC region as of 2017 although its screening rates greatly varies between LAC countries [27]. While, as an instance, Cuba and Belize screening rates have reached a 100 % coverage, Haiti only reached 35 % coverage [22]. In 2015, Cuba became the first country in the world to receive validation from the WHO for having achieved elimination of MTCT of syphilis and HIV [28]. In 2017, the Caribbean islands of Anguilla, Antigua and Barbuda, Bermuda, Cayman Islands, Montserrat and Saint Kitts and Nevis followed Cuba in this success [28]. Despite that, other LAC countries are seeing an increase in the prevalence of syphilis among pregnant women. Recent literature suggests that syphilis prevalence is escalating in Chile, contributing to an increase of adverse pregnancy outcomes [29]. Similarly, a study in Argentina showed an increased prevalence of *Chlamydia trachomatis* among pregnant women under 25 years old with low socioeconomic status, calling for routine screening of this pathogen at least among this population group [30]. Currently, it is difficult to assess the real burden of chlamydia, gonorrhea, trichomonas, and even more mycoplasma among pregnant women in the LAC region, as no routine screening for these STIs is mandatory and literature is limited and not updated [4].

Given the previously mentioned effects on maternal and child health together with the high burden of these STIs in LAC, its screening during pregnancy should be considered especially among high-risk population groups. Furthermore, several studies already highlighted the benefits of screening chlamydia or syphilis infection among pregnant women with or without high risk of infection *vs* non-screening for these STIs [[31], [32], [33]]. Additionally, full coverage of syphilis screening should be accomplished in all LAC countries. The prevalence of, and likely adverse outcomes associated with these curable non-viral STIs in pregnant women suggests that etiologic STI screening followed by targeted treatment might be beneficial during pregnancy [7]. To our knowledge, no study has comprehensively reviewed the association between non-viral STIs and adverse pregnancy outcomes in Latin America and the Caribbean. In this review, our aim was to report on the association between curable non-viral STIs and pregnancy outcomes among women living in the LAC region. Results from this study will inform future research by contributing evidence needed to support developing robust guidelines to improve maternal and child health conditions for the LAC region.

## 2 Methods

A systematic literature search was performed following the Preferred Reporting Items for Systematic Review and Meta-Analysis Statement (PRISMA) guidelines [34]. The corresponding completed PRISMA form can be found in S1 Checklist.

### 2.1 Search strategy

Published studies assessing the impact of adverse pregnancy outcomes related with non-viral STIs were eligible for this review up to August 22nd, 2022. The Pubmed/Medline, Embase (Ovid), SciELO and LILCAS electronic databases were used to conduct the literature search. The SciELO database was utilized as it is a commonly used bibliographic database in the LAC region enclosing 14 LAC countries which publish their journal collections in English, Portuguese and Spanish [35]. Similarly, LILACS (*Literatura Latinoamericana y del Caribe en Ciencias de la Salud* or Latinoamerican and Caribbean Literature in Health Sciences) is a database which encloses all research articles published by 21 LAC countries [36]. The Medical Subject Heading, Embase subject heading (Emtree) terms, SciELO and LILACS search strategies, free-text terms and combinations of these keywords are compiled in S2 Table.

The following keywords were used for the literature search: “sexually transmitted infections”, “non-viral”, “adverse pregnancy outcome”, “Latin America”, “Caribbean”, “South America”, “Latin America and the Caribbean”, “pregnant women”, “pregnancy” and “pregnancy outcome”. To ensure that all of the available studies were included in the review, the terms “Latin America”, “Caribbean”, “South America” and “Latin America and the Caribbean” were switched to the individual name of the 42 Latin American and Caribbean countries (as defined by the World Bank [37]). Finally, articles with information about non-viral STIs and pregnancy outcomes found on the reference list from the extracted studies were examined to retrieve potential articles via snowballing.

### 2.2 Eligibility criteria

The following inclusion criteria were used: (1) studies written and/or translated in English or Spanish languages; (2) cross-sectional/prevalence, case-control or prospective and retrospective cohort study designs; (3) studies conducted in Latin America and/or the Caribbean; (4) including pregnant women with known perinatal outcomes exposed to non-viral STIs before and/or during pregnancy and (5) diagnosis for non-viral STI performed on samples collected during pregnancy and up to 48 h after the newborns’ birth. Studies were excluded if (1) the women received treatment intrapartum for the non-viral STI, except for syphilis; (2) if there was not a control group present in the study and (3) if diagnosis of the pathogen was not performed using laboratory diagnostic tools on samples collected up to 48 h after delivery.

### 2.3 Outcome measurements

The following late adverse pregnancy outcomes, based on the WHO definition, were reported on: preterm birth (<37 weeks 0 days gestation [38]), low birth weight (<2500g [39]), spontaneous abortion or stillbirth (delivery of stillborn infant ≥20 weeks 0 days [40]) and neonatal death (death within first 28 days of life [41]).

### 2.4 Study selection

Articles were assessed by means of their title and abstract by one researcher (MLE). The full text of potential articles was read and assessed for their eligibility according to the eligibility criteria. In cases of ambiguity, two additional researchers (CG and EA) were consulted. Additionally, bibliographies of potentially relevant papers, even if they were excluded during the selection process, were screened for additional potential studies.

### 2.5 Summary measures

Country, type of STI and adverse pregnancy outcome, type of sample collected and when, diagnostic tool used, number of pregnant women included per infected and control group, gestational age at testing, mean or median's mother and the adjusted Odds Ratio (OR) and 95 % confidence intervals (CI) were extracted or calculated (based on the dichotomous data given). Forest plots depicting ORs were performed using R Studio 3.6.3 vesion [42]. Meta-analysis was performed when two or more studies with the same study design reported the same outcome.

### 2.6 Assessment of methodological quality of included studies

The methodological quality of the included studies was assessed following the guidelines of the Joanna Briggs Institute (JBI) reviewer's manual [43]. For each single study, a respective JBI checklist, made for different study designs and containing questions to assess the potential risk of bias, was applied (S3 Table). To calculate the risk of bias, every question was answered with “yes” or “no” or “unclear”. For “yes”, 1 point was assigned, for “no” 0 points and for “unclear” 0.5 points. The total amount of points divided by the number of questions multiplied by 100 gave the percentage of risk of bias. A score of 70 % or higher indicated a low risk of bias, a score between 50 and 69 %, a moderate risk of bias and a score below 50 % showed a high risk of bias.

## 3 Results

### 3.1 Study selection

**Fig. 1:**
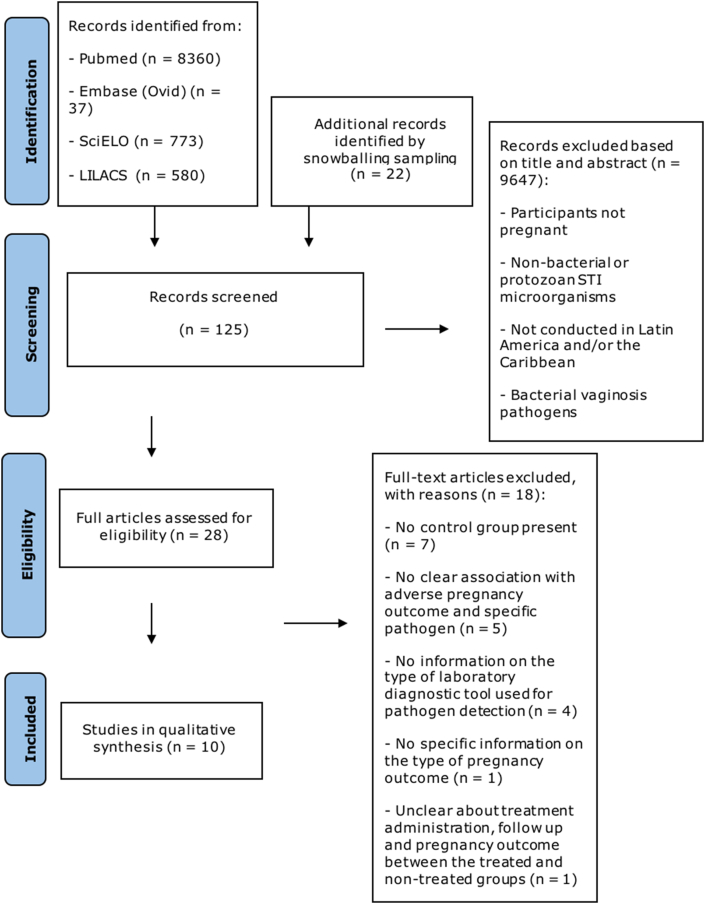
Prisma Flow Diagram Prisma Flow Diagram for Inclusion and Exclusion of Studies Extracted and adapted from Ref. [34].

The search strategy in this review yielded 8360 records from Pubmed, 773 from SciELO, 580 from LILACS, 37 from Embase (Ovid) and 22 through snowballing search. The study selection process is shown in the PRISMA from diagram (Fig. 1). Among them, 10 met the inclusion criteria [15,[43], [44], [45], [46], [47], [48], [49], [50], [51]]. From the included articles, 6 were retrieved from Pubmed (4 via specific LAC country name search strategy and 2 via general search strategy); 2 via SciELO database and 2 via snowballing search. Although some articles written in Spanish were assessed for eligibility, only articles in English fulfilled the inclusion criteria and were included. The selected studies reported pregnant women living in Latin America or the Caribbean, who presented active laboratory-confirmed infection by the causative agents, *Treponema pallidum, Chlamydia trachomatis* and *Mycoplasma genitalium*.

### 3.2 Population and study characteristics

**Fig. 2:**
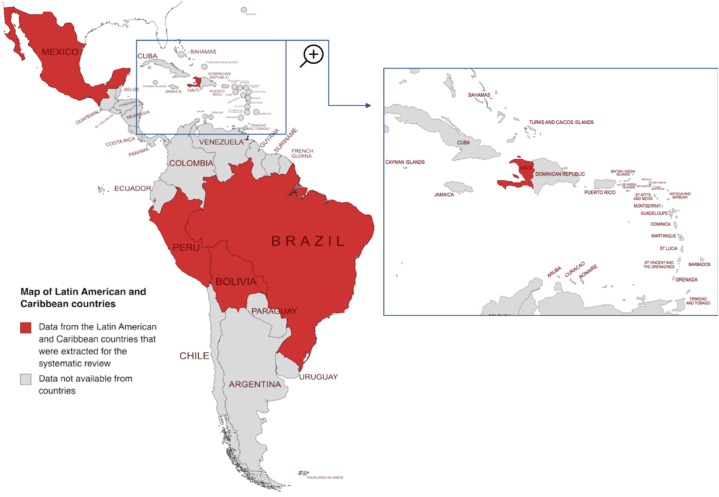
Map of Latin America and the Caribbean depicting in red the countries where the included studies were conducted. Map depicting studies from five LAC countries (Bolivia, Brazil, Haiti, Mexico and Peru) that were retrieved. Map created and edited from Mapchart [52] (For interpretation of the references to colour in this figure legend, the reader is referred to the Web version of this article.)

In total, studies from five LAC countries (Bolivia, Brazil, Haiti, Mexico and Peru) were retrieved (Fig. 2). Six cross-sectional studies, three cohort studies and one case-control study were included. Supplementary Table 4 (S4 Table) provides a summary of the cohort characteristics and methodological features of the retrieved studies. Pregnant women's (mean or median) age was determined in 8 out of the 10 chosen studies (S4 Table) [15,44] [[15], [44], [45], [46], [47], [48], [49], [50], [51]] [[15], [44], [45], [46], [47], [48], [49], [50], [51]]. From the included studies, Benedetti et al. reported the oldest pregnant women's age of 26 years old (mean) [45].

Sample collection was performed during prenatal care in the majority of the articles [[44], [45], [46],^48^,^51^,^53^], although some proceeded at delivery [47,50,54] or up to 48 h postpartum [15] when women were still admitted in the hospital. All included articles confirmed STI by laboratory techniques. For *Treponema pallidum* diagnosis on blood samples, Araújo et al., Cardoso et al. and Benedetti et al. used Venereal Disease Research Laboratory (VDRL) non-treponemal testing [44,45,55]. Cardoso et al. followed with an enzyme-linked immunosorbent assay (ELISA) for confirmation of active syphilis infection. Southwick et al., Guarner et al. and Behets et al. diagnosed syphilis with rapid plasma reagin (RPR) testing following with a treponemal antibody absorption test [50,51,54]. Endocervical sampling was the method of choice for sample collection for all chlamydia and mycoplasma studies, except for Schmidt et al., which decided to diagnose *Chlamydia trachomatis* on urine samples [47]. DNA extraction and polymerase chain reaction (PCR) were conducted for diagnosing *Chlamydia trachomatis* infection on Casillas-Vega et al., Schmidt et al. and de Borborema-Alfaia et al. [[46], [47], [48]]. For *Mycoplasma genitalium* infection, Hitti et al. conducted cervicovaginal sampling where authors performed *Mycoplasma genitalium* PCR testing [15].

Table 1 encompasses the documented findings from studies that have examined the relationship between non-viral sexually transmitted infections (STIs) and pregnancy outcomes.

**Table 1:** The association between syphilis, chlamydia and mycoplasma infections with adverse pregnancy outcome in women living in Latin America and the Caribbean.

Author, year	Country	N positive women for STI	N women/newborn STI+ and adverse pregnancy outcome	Odds ratio (OR)		95 % CI, *P*-value (p)
*Treponema pallidum* and premature birth						
Araújo et al., 2021 [44]	Brazil	134/478 TP + women	73 premature newborns TP+		**OR=3.29**	**95 % CI** **1.93**–**5.61, p**<**0.0001**
***Treponema pallidum* and history of previous spontaneous abortion**						
Behets et al., 1995 [51]	Haiti	110/996 TP + women	Data not shown in study		**OR=1.72**	**95 % CI** **1.04**–**2.84, p=0.02**
Benedetti et al., 2019 [45]	Brazil	29/661: (21/661 TP + at antenatal; 4/661 TP + at delivery; 4/661 TP + at postpartum)	14/29 TP+ with history of previous spontaneous abortion		**OR=3.7**	**95 % CI** **1.7**–**8, p**<**0.01**
***Treponema pallidum* and fetal and infant death**						
Cardoso et al., 2015 [53]	Brazil	3142 TP + women	382 total cases: 309 fetal deaths and 73 infant deaths related to CS		**OR=3.2**	**95 % CI** **1.54**–**6.62, p=0.02**
***Treponema pallidum* and low birth weight**						
Southwick et al., 2001 [50]	Bolivia	61/1428 TP + women	7 TP + infants with low birth weight		**OR=2.6**	**95 % IC** **1.2**–**4.8, p**<**0.001**
***Treponema pallidum* and funisitis**						
Guarner et al., 2000 [54]	Bolivia	66/1559 TP + women	9 TP + newborns with funisits		**OR=41.2**	**95 % CI** **5.5**–**405.7, p**<**0.001**
***Chlamydia trachomatis* and premature birth**						
Schmidt et al., 2015 [47]	Brazil	45/323 CT + women	17/323 CT + premature newborns		OR = 1.4	95 % CI 0.6–3.1, p = 0.404
Casillas-Vega et al., 2017 [46]	Mexico	96/662 CT + women	12/96 CT + premature newborns		**OR=1.98**	**95 % CI** **0.96**–**3.88, p=0.03**
***Chlamydia trachomatis* and ectopic pregnancy**						
Casillas-Vega et al., 2017 [46]	Mexico	96/662 CT + women	4/96 CT + ectopic pregnancies		**OR=3.46**	**95 % CI** **0.87**–**12.19, p=0.03**
***Chlamydia trachomatis* and respiratory symptoms**						
de Borborema-Alfaia et al., 2013 [48]	Brazil	11/88 CT + women	8 CT + newborns with respiratory symptoms		a**OR=34.5**	**95 % CI** **6.22**–**191.46, p**<**0.05**
***Mycoplasma genitalium* and premature birth**						
Hitti et al., 2010 [15]	Peru	41/661 MG + women	29 premature newborns		**OR=2.4**	**95 % CI** **1.1**–**5.3, p=0.03**

Forest plots depicting the association between non-viral STIs (syphilis, chlamydia and mycoplasma) and adverse pregnancy outcomes in pregnant women living in Latin America and the Caribbean. *P*-values indicate differences in outcomes between pregnant women with diagnosed STIs and without infection in individual studies (statistically significant differences in bold = p < 0.05). Data are expressed as OR. OR = 1 is indicated as a dashed grey line in the forest plots.

a Calculated based on the number given in the original paper. CI, confidence interval. CT, *Chlamydia trachomatis*. CS, congenital syphilis. MG, *Mycoplasma genitalium*. OR, odds ratio. TP, *Treponema pallidum*.

### 3.3 Syphilis and adverse pregnancy outcomes

Regarding syphilis infection, 6 studies reported five types of adverse pregnancy outcomes, namely preterm birth, history of previous spontaneous abortion, fetal and infant death, low birth weight and funisitis of the umbilical cord [44,45,[50], [51], [54], [55]].

Araújo et al. investigated the association between *Treponema pallidum* infection and preterm birth in Brazil. The authors defined preterm birth as the delivery of the newborn prior to 37 weeks of gestation. Araújo and co-authors unraveled that the cases of preterm birth observed among *Treponema pallidum* positive pregnant women occurred in women who were not treated or four women that received some drug other than penicillin, increasing the outcome in the population that received no or incomplete treatment [44]. Similarly, only 4 out of the 166 syphilis positive women were considered to be fully treated and did not present any adverse pregnancy outcome related to this infection. A significant association was found between syphilis and preterm birth (Table 1). Based on this, pregnant women who were syphilis-positive had a 3.3-fold increase in the incidence of preterm birth compared to non-infected mothers (95 % CI 1.93–5.61).

Behets et al. and Benedetti et al. looked at the association between syphilis infection and history of previous spontaneous abortion [44,49]. The two studies, the first one conducted in Haiti and the second one in Brazil, reported an association between syphilis and history of spontaneous abortion with ORs ranging from 1.7 to 3.7^52,57^.

Cardoso et al. investigated the association between *Treponema pallidum* infection and fetal and infant death [55]. Neonatal and infant death was defined as death occurred from 22 gestational weeks to the 7th day of life. This study reported an association between syphilis-infected women presenting neonatal and infant death among their newborns with signs of congenital syphilis (OR = 3.2, 95 % CI 1.54–6.62).

The association between low birth weight and *Treponema pallidum* infection was investigated by Southwick et al. [50]. Newborns whose mothers were syphilis positive, were 2.6-fold more likely to present low birth weight (median of 2200 g) (OR = 2.6; 95 % IC 1.2–4.8).

Guarner et al. evaluated the association between syphilis infection and funisitis of the umbilical cord, as a cohort study conducted in Bolivia [54]. The presence of treponemes in the umbilical cord from syphilis-positive women was determined at postpartum (≥37 weeks) stage. An association was found with syphilis infected women and funisitis (OR = 2.6, 95 % CI 1.2–4.8).

### 3.4 Chlamydia and adverse pregnancy outcomes

For chlamydia infection, 3 studies of late adverse pregnancy outcomes were included in the systematic review. These studies specifically assessed history of preterm birth, ectopic pregnancy and respiratory symptoms on the newborn [[45], [46], [47]].

Casillas-Vega et al. in Mexico, and Schmidt et al. in Brazil, investigated the association of *Chlamydia trachomatis* infection and preterm birth [46]. Casillas-Vega and co-authors concluded that women who were positive for chlamydia had 1.98-fold higher risk of presenting history of premature births (Table 1). Schmidt et al. settled the definition of preterm birth as any infant born between 22 weeks and 36 weeks and 6 days before delivery time. Nevertheless, they found no significant association between *Chlamydia trachomatis* infection and preterm birth (OR = 1.4, 95 % CI 0.6–3.1).

Casillas-Vega et al. also studied the association between *Chlamydia trachomatis* infection among pregnant women with history of ectopic pregnancy [46]. From a cohort of 662 pregnant women, 96 presented *Chlamydia trachomatis* infection and from them, 4 ectopic pregnancies were confirmed (OR = 3.5, 95 % CI 0.87–12.9).

Respiratory symptoms on the newborn, such as cough, nasal obstruction and dyspnea were associated with *Chlamydia trachomatis* infection by de Borborema-Alfaia et al. in a cross-sectional study performed in Manaus, in the Brazilian Amazon region [48]. From a cohort of 88 pregnant women, 11 presented laboratory confirmed chlamydia infection, where 8 of their newborns had respiratory symptoms (OR = 34.5, 95 % CI 6.22–191.46).

### 3.5 Mycoplasma and adverse pregnancy outcome

The association between *Mycoplasma genitalium* infection and adverse pregnancy outcome was assessed by one case-control study conducted in Peru by Hitti et al. [15]. The case group included 661 pregnant women who suffered from spontaneous premature birth from week 20 until week 36 of pregnancy. As a control group, 667 women who delivered ≥37 weeks were also screened for the STI. In the case group, *Mycoplasma genitalium* was present in 29 preterm newborns, whereas only 9 newborns were diagnosed with *Mycoplasma genitalium* in the control group (OR = 2.4, 95 % CI 1.1–5.3).

### 3.6 Risk of bias within studies

All cross-sectional, case-control and cohort studies were at risk of selection bias as described by the JBI critical appraisal tool (S5 Table). De Borborema-Alfaia et al. and Behets et al. studies had moderate risk of bias and the remaining 8 included studies had low risk of bias. De Borborema-Alfaia et al., a cross-sectional study about *Chlamydia trachomatis* infection and pregnancy outcome in Brazil had a score of 68.8 % for risk of selection bias. For a cross-sectional study, the setting and participant's selection lacked further explanation. Questions five and six were also lacking as this study did not assess confounding factors - age, number of sexual partners, education, etc. - neither in the *Chlamydia trachomatis* positive and negative study cohorts. When performing statistical analysis, authors lacked in adjusting for confounding factors, as they were never taken into consideration. Finally, the discussion could have been enriched by the inclusion of these points, nevertheless they were missed. Behets et al., a cohort study associating *Treponema pallidum* infection with adverse pregnancy outcomes in Haiti, scored a 65 %, therefore a moderate risk of bias. Contrary to de Borborema-Alfaia et al., Behets and co-authors assessed confounding factors and performed statistical analysis adjusting all the variables in both groups (pregnant women with syphilis *vs* pregnant women syphilis negative). Nevertheless, this study failed in assessing the outcome variable (spontaneous abortion) in a valid and reliable way, and mainly because of this, it scored moderate risk of selection bias.

## 4 Discussion

To our knowledge, this is the first study to comprehensively review and synthetize the association between non-viral STIs and pregnancy outcome among women living in Latin American and Caribbean countries. To identify all relevant work on the topic, studies including adverse effects on pregnancy outcome due to infections from all possible non-viral STIs were systematically identified and evaluated. Ten studies were included in our review as associated *Treponema pallidum, Chlamydia trachomatis* and *Mycoplasma genitalium* with adverse pregnancy outcomes namely preterm birth, history of spontaneous abortion, fetal and infant death, low birth weight, funisitis of the umbilical cord, ectopic pregnancy and respiratory symptoms on the newborn [15,[43], [44], [45], [46], [47], [48], [49], [50], [51]].

Six studies assessed the association between active, non-treated *Treponema pallidum* infection and adverse pregnancy outcome, meaning preterm birth, previous spontaneous abortion, fetal and infant death low birth weight and funisitis of the umbilical cord [44,45,50,51,54,53]. These results are in line with other published data from the LAC region [29,55,56,57]. In Haiti, a significant relationship between adverse pregnancy outcomes (stillbirths and neonatal deaths) with maternal syphilis status (p < 0.0001) was found in rural pregnant women [56]. Similarly, Elarrat-Canto et al. described how congenital syphilis accounted for 87.7 % and 79.3 % of total perinatal and fetal deaths reported to the Brazilian Notifiable Diseases Information System from 2010 to 2014 [55]. A stagnant, or even increased trend of syphilis in the newborn is being observed generally among all LAC countries, as mandatory syphilis screening during pregnancy is not widespread [26]. The Pan American Health Organization estimated that in Brazil, the vertical transmission rate of syphilis doubled between 2010 and 2015, contributing to an increased rate of congenital syphilis disease [58]. Seabra et al. also observed an increased trend of congenital syphilis from 2015 to 2018 mainly due to gaps on health coverage during prenatal care [59]. Similarly, in Chile, syphilis rates among pregnant women increased from 0.4 to 7.2 per 1000 live births from 2013 to 2019 [29]. In Uruguay, congenital syphilis rates increased from 1.6 cases among 1000 births in 2015 to 9.5 cases among 1000 births in 2019 where the majority of the cases were related to uncontrolled pregnancies due to the women's low socioeconomic status and difficulties accessing health care [60]. Furthermore, different trends in syphilis coverage are observed between LAC countries. Whereas in Argentina, it is reported that syphilis screening coverage reached 95.3 % from 2010 to 2013, in Guatemala, syphilis screening coverage only reached 44.8 %, similar screening coverage as in India (46.7 %), and significantly lower than in Kenya (79.8 %) or Zambia (85.1 %) [57]. As described throughout the review, it is known that syphilis is associated with diverse adverse pregnancy outcomes. *Treponema pallidum* screening should be strongly advised among all LAC countries, promoting that the highest number of pregnant women are tested during prenatal care. A cost-effectiveness study including 20 LAC countries reported that antenatal syphilis screening is highly cost-effective in Latin America and the Caribbean as adverse pregnancy outcomes can be averted in areas of high risk of infection [61]. As recently described on a review about syphilis testing techniques among pregnant women, the main disadvantage of syphilis screening is that the use of treponemal and non-treponemal tests for syphilis diagnosis requires laboratory capacity and trained personnel [62]. In resource-limited settings in LAC regions, the use of point of care testing (POCT) could be implemented during antenatal care for syphilis testing [[62], [63], [64]]. As an instance, in Bolivia, the use of POCT has proven to be more sensitive than routine RPR and had comparable specificity when performing syphilis diagnosis among pregnant women [65]. In this sense, POCT could be a way to expand syphilis screening to clinics with no laboratory facilities, improve case detection and facilitate treatment delivery [65]. Appropriate treatment scheme according to the stablished protocol and technical note (appropriate drug, dosage and interval) should also be considered when diagnosing a positive case of syphilis during pregnancy [66]. In a study conducted in the Brazilian Amazonian region, approximately 60 % of the women diagnosed with syphilis were treated with single dose penicillin, considered insufficient for controlling congenital syphilis, being able to still transmit the infection to their infants [67]. As with inadequate treatment, it should be remarked that both the partners' treatment and the treatment monitoring for pregnant women should be addressed and controlled during antenatal care [66]. In Haiti, Lomotey et al., confirmed that all positive syphilis postpartum women had evidence of treatment for the disease, nevertheless the time of gestation when women were treated was available for only 24 of the 31 positive women and 11 % of them received inadequate treatment against syphilis (meaning non-penicillin therapy given less than 30 days before delivery) [56]. In this context, as outlined by Nonato et al., it is common to find records assessing the lack of information about the women's treatment for *Treponema pallidum* infection [68]. Gestational age, stage of the disease or prescribed dosage, or even the time elapsed between the end of the treatment and the childbirth are data which tends to be underreported, as well as the underestimation or misinformation about partner's treatment which may progress to syphilis re-infections during pregnancy [68]. As an instance, achievements can be accomplished, as Cuba eliminated MTCT of syphilis by developing an initiative which ensured prompt and free-of-charge access to prenatal care, free and reliable testing for both women and their partners among all population groups, offering adequate treatment for infected women and their newborns [69].

*Chlamydia trachomatis* infection has been associated with adverse pregnancy outcomes, namely history of preterm birth, ectopic pregnancy, and respiratory symptoms on the newborn,^53–55^. In Latin America and the Caribbean, several studies also support these results [[70], [71], [72], [73]]. In Brazil and Argentina, Adachi et al. associated *Chlamydia trachomatis* infection with low birth weight and preterm delivery (p = 0.008) among pregnant women co-infected with HIV and *Neisseria gonorrhoeae* [70]. In Curaçao, Hage et al., demonstrated that patients with preterm delivery were highly co-infected by *Chlamydia trachomatis* and *Neisseria gonorrhoeae* pathogens, although no association was made in the study population [73]. Similarly, a case-control study among Chilean pregnant women conducted STI screening of *Chlamydia trachomatis, Neisseria gonorrhoeae* and *Trichomonas vaginalis* in asymptomatic and symptomatic women [71]. *Chlamydia trachomatis* infection was found on 12.9 % of pregnant women and 2.4 % presented co-infection with *Trichomonas vaginalis,* but not with *Neisseria gonorrhoeae* [71]. Although chlamydia has been clearly associated with adverse pregnancy outcomes in infected mothers, no current programs conduct antenatal screening in Latin America and the Caribbean, except in the Bahamas [72]. Several studies unravel the cost-benefit of performing chlamydia infection screening during prenatal care in high-income countries as the United States [74] and the Netherlands [75]. A recent review focusing in LMICs outlined the feasibility of performing chlamydia infection screening during pregnancy, given the fact that in general, LMICs present considerably high prevalence rates of this infection. Similarly, Cabeza et al., conducted a prospective study where they successfully assessed the cost-benefit of performing *Chlamydia trachomatis* screening among pregnant women attending antenatal care clinics in two urban hospitals in Lima, Peru [72]. As screening for *Chlamydia trachomatis* infection has been proven to be feasible [76], its implementation could be promoted, specially (1) among young high-risk pregnant women attending prenatal care and (2) living in LAC regions where laboratory techniques can be conducted to diagnose the infection. Additionally, it is imperative to consider the comprehensive assessment of risk factors associated with chlamydial infection. Identification and understanding of these risk factors can aid in the development of effective screening protocols and targeted interventions. Individual risk factors include demographic characteristics such as young age, as well as certain behavioral patterns, including early sexual debut, multiple sexual partners, and inconsistent or non-use of contraceptive methods. Furthermore, a history of previous STI, has been found to elevate the risk of recurrent infections [77]. Contextual factors also play a significant role in chlamydia transmission dynamics. Intimate partner-related factors, such as having a partner with a history of STIs or concurrent sexual relationships, have been strongly associated with increased chlamydial infection rates. Hence, a comprehensive screening approach should not only focus on the pregnant woman but also emphasize the importance of partner screening, notification, and treatment strategies. Partner involvement is crucial in preventing reinfection and interrupting transmission chains, as it can contribute to the success of treatment and eradication efforts [78]. Recognizing the significance of these risk factors and their interplay, it is essential for clinicians to adopt a multifaceted approach to chlamydia screening in high-risk pregnant women.

Very little information on the prevalence of *Mycoplasma genitalium* infection among pregnant women is reported in the LAC region [8]. A study conducted in Argentina found a high prevalence of infection of 5.2 % among pregnant women attending prenatal care in a public hospital in Buenos Aires [5]. In this review, one case-control study conducted in Peru positively associated *Mycoplasma genitalium* infection with preterm birth [15]. Only few studies have investigated the role of *Mycoplasma genitalium* and preterm birth, as an instance in the United States [79], Japan [80] or in Guinea Bissau [81] where no association between this pathogen and adverse pregnancy outcome was found, despite the authors called for further investigation. In this context, more research is needed to stablish definitive associations between *Mycoplasma genitalium* and adverse pregnancy outcomes in order to promote screening recommendations during pregnancy.

Several studies focusing on non-viral STIs and pregnancy outcome outlined how belonging to certain population and socioeconomic groups are barriers to accessing antenatal care and therefore, suffering from a higher prevalence of STIs [82,58,[83], [84]]. The LAC region, which faces high rates of social inequalities, health inequities remain extremely visible when addressing reproductive, maternal and child health interventions among the 3 main ethnic groups – indigenous people, African descendant people and people of European descent [85,86]. Several studies included in this review also addressed socioeconomic and ethnic inequalities with STI prevalence [44,45,48,49,59,60]. Araújo et al., reported associations between *Treponema pallidum* and prematurity in Brazilian women who did not have a paid job (95 % CI 0.97–3.46), were drug users (95 % CI 1.39–4.11) and were not able to attend prenatal care (95 % CI 1.28–4.05) [44]. Southwick et al., concluded that pregnant women who had less than secondary-level education (95 % CI 2.0–6.8), had lower socioeconomic status (95 % CI 1.2–3.4) or had more than one partner during pregnancy (95 % CI 1.5-38-7) were more at risk for delivering newborns with syphilis [50]. In Argentina, syphilis seroprevalence was found to be 8.71 % among the Mbya Guarani indigenous communities, whereas the prevalence of syphilis among the general Argentinian population was considered to be 0.8 % [87,88]. In Cali, Colombia, Benítez et al. estimated that 74 % of congenital syphilis cases were diagnosed among the population in situation of economic vulnerability, where 25 % of the cases occurred among the African-Americans [89,90]. Economic, geographic and cultural barriers prevent regular access to basic health services, affecting the frequency of STI testing and consequently, its treatment and pregnancy outcome among at-risk vulnerable population groups [86,87].

## 5 Strengths and limitations

The broad search strategy, using four different well-known and region-specific databases is a strength of this review. It allowed for identification of a wide range of different studies and it is unlikely that large papers were missed. The defined inclusion and exclusion criteria allowed a clear selection process for the included studies. Moreover, duplicate screening prevented data entry errors and risk of bias assessment of the included articles averted the inclusion of biased studies. By including studies only conducted in Latin America and the Caribbean, which did not address other pathogen than non-viral STIs, we ensured that the main focus of the review was to emphasize the role of non-viral STIs, which are often eclipsed by viral STIs, and its association with pregnancy outcome in the region. This is also a limitation as, given the established associations between non-viral STIs and adverse pregnancy outcomes, curable non-viral STIs are not given as much public health attention as other STIs. Therefore, although relevant articles were found and included in the review, there are not many studies addressing non-viral STIs and its association with adverse pregnancy. Finally, although studies regarding curable non-viral STIs like *Neisseria gonorrhoeae* and *Trichomonas vaginalis* infections during pregnancy were screened and assessed for eligibility, none fulfilled the inclusion criteria to be included in the review.

## 6 Conclusion and future perspectives

This is the first study addressing the association of non-viral STIs, in particular *Treponema pallidum, Chlamydia trachomatis* and *Mycoplasma genitalium* among pregnant women reporting adverse pregnancy outcomes in Latin American and Caribbean countries. Most of the time, pregnant women with low socioeconomic and educational positions are left without proper antenatal coverage care, especially in LMICs. In the LAC region, as socioeconomic inequalities are visibly present, inequities in maternal and child health coverage are considerably observed across different population groups. It is important to emphasize the need for an equitable distribution of resources in health services as the burden of non-viral STIs is escalating among high-risk and disadvantaged population groups in the Latin American and Caribbean region.

Given the well-known associations of non-viral STIs with adverse pregnancy outcomes, this review is a call, together with other published studies, to consider the implementation of public health programs in Latin America and the Caribbean to (1) increase syphilis screening and treatment coverage among pregnant women and their partners; (2) to implement antenatal screening programs using laboratory-based methods for detecting *Chlamydia trachomatis* infection during pregnancy, at least in high-risk population groups and (3) to promote further research on the association of other non-viral STIs (*Mycoplasma genitalium, Neisseria gonorrhoeae* and *Trichomonas vaginalis*) on pregnancy outcomes.

## Funding statement

The research received no specific funding from any agency in the public, commercial, or not-for-profit sectors.

## Additional information

No additional information is available for this paper.

## Data availability statement

All data cited in this systematic review is publicly available and can be accessed online through the respective publishers, repositories, or databases. The complete list of cited papers, along with their sources, is provided in the reference section of this manuscript. Additionally, detailed information, supplementary materials, and relevant data supporting the findings of this review are available in the accompanying appendix.

## CRediT authorship contribution statement

**Maria Lara Escandel:** Writing – original draft, Methodology, Formal analysis, Data curation. **Carlotta Gamberini:** Writing – review & editing, Supervision. **Naomi C.A. Juliana:** Conceptualization. **Salwan Al-Nasiry:** Writing – review & editing, Supervision. **Servaas A. Morré:** Writing – review & editing. **Elena Ambrosino:** Writing – review & editing, Supervision, Conceptualization.

## Declaration of competing interest

The authors declare the following financial interests/personal relationships which may be considered as potential competing interests:Servaas A. Morre’ reports a relationship with inBiome that includes: equity or stocks. Servaas A. Morre’ reports a relationship with Microbe&Lab BV that includes: equity or stocks.
