# Supplementary material for: The association between non-viral sexually transmitted infections and pregnancy outcome in Latin America and the Caribbean: A systematic review
Source: Heliyon. 2023 Dec 13;10(1):e23338. doi: 10.1016/j.heliyon.2023.e23338 (PMC10767377; doi:10.1016/j.heliyon.2023.e23338)
Supplement: Multimedia component 1 [file mmc1.docx]

Supplementary material

**S1 Table.** Overview of non-viral sexually transmitted infections (STIs) included in the systematic review.

| **STI** | **Type of causative agent** | **Associated adverse pregnancy outcome(s)** | **Laboratory diagnostic tools to detect pathogen** | **Screening during pregnancy in the LAC region** |
| --- | --- | --- | --- | --- |
| Syphilis | *Treponema pallidum,*  Spirochete bacteria | Early fetal loss, stillbirth, prematurity, low birth weight, neonatal death and congenital syphilis disease | Serologic non-treponemal tests (RPR and VDRL), treponemal tests (FTA-ABS, MHA-TP and EIA) and rapid POCT | Strongly advised among all Latin American and Caribbean countries |
| Chlamydia | *Chlamydia trachomatis,*  Intracellular bacterium | Premature rupture of membranes, preterm birth, low birth weight, stillbirth and infections on the newborn | Conventional NAATs and PCR based RDTs | Can be required for pregnant women presenting symptoms for STI combined with syndromic management |
| Gonorrhea | *Neisseria gonorrhoeae,*  Gram-negative bacteria | Preterm birth and newborn infections | Light microscopy and NAATs (including POCTs) | Can be required for pregnant women presenting symptoms for STI combined with syndromic management |
| Trichomoniasis | *Trichomonas vaginalis,*  Parasitic protozoan | Preterm birth | Wet preparation microscopy, cell culture and NAATs (including POCTs) | Can be required for pregnant women presenting symptoms for STI combined with syndromic management |
| Mycoplasma | *Mycoplasma genitalium,*  Intracellular bacterium | Preterm birth | NAATs | Not required |

EIA, enzyme immunoassay. FTA-ABS, treponemal antibody absorbed test. MHA-TP, microhemagglutination assay for *Treponema pallidum*. NAAT, nucleic acid amplification test. PCR, polymerase chain reaction. POCT, point of care testing. RDT, rapid diagnostic test. RPR, rapid plasma reagin. VDLR, venereal disease research laboratory. References^10-15,19,20^.

**S2 Table.** Search strategies and hits for searches that were conducted.

| **Database** | **Search strategy** | **Hits** |
| --- | --- | --- |
| Pubmed | General research strategy:  (("reproductive tract infections"[Mesh]) OR ("sexually transmitted diseases"[Mesh]) OR (genital tract infection) OR (reproductive tract infection) OR (sexually transmitted infection) OR (sexually transmitted diseases) OR (vaginal infection)) AND (("pregnant women"[Mesh]) OR ("pregnancy"[Mesh]) OR (pregnancy) OR (pregnant women)) AND (("pregnancy outcome"[Mesh]) OR (adverse pregnancy outcome) OR (pregnancy outcome)) AND (("South America and the Caribbean"[Mesh]) OR ("Latin America and the Caribbean"[Mesh]) OR (Latin America) OR (the Caribbean) OR (South American women) OR (Latin American women) OR (Caribbean women))  Specific country search strategy:  (("reproductive tract infections"[Mesh]) OR ("sexually transmitted diseases"[Mesh]) OR (genital tract infection) OR (reproductive tract infection) OR (sexually transmitted infection) OR (sexually transmitted diseases) OR (vaginal infection)) AND (("pregnant women"[Mesh]) OR ("pregnancy"[Mesh]) OR (pregnancy) OR (pregnant women)) AND (("pregnancy outcome"[Mesh])  OR (adverse pregnancy outcome) OR (pregnancy outcome)) AND (("Brazil"[Mesh]) OR ("Mexico"[Mesh]) OR ("Colombia"[Mesh]) OR ("Argentina"[Mesh]) OR ("Peru"[Mesh]) OR ("Venezuela"[Mesh]) OR ("Chile"[Mesh]) OR ("Guatemala"[Mesh]) OR ("Ecuador"[Mesh]) OR ("Bolivia"[Mesh]) OR ("Haiti"[Mesh]) OR ("Cuba"[Mesh]) OR ("Dominican Republic"[Mesh]) OR ("Honduras"[Mesh]) OR ("Paraguay"[Mesh]) OR ("Nicaragua"[Mesh]) OR ("El Salvador"[Mesh]) OR ("Costa Rica"[Mesh]) OR ("Panama"[Mesh]) OR ("Uruguay"[Mesh]) OR ("Jamaica"[Mesh]) OR ("Trinidad and Tobago"[Mesh]) OR  ("Guyana"[Mesh]) OR ("Suriname"[Mesh]) OR ("Belize"[Mesh]) OR ("Bahamas"[Mesh]) OR ("Barbados"[Mesh]) OR ("Saint Lucia"[Mesh]) OR ("Grenada"[Mesh]) OR ("St. Vincent and Grenadines"[Mesh]) OR ("Antigua and Barbuda"[Mesh]) OR ("Dominica"[Mesh]) OR ("Saint Kitts and Nevis"[Mesh]) OR ("Aruba"[Mesh]) OR ("British Virgin Islands"[Mesh]) OR ("Cayman Islands"[Mesh]) OR ("Curacao"[Mesh]) OR ("Dominica"[Mesh]) OR ("Sint Maarten"[Mesh]) OR ("St Lucia"[Mesh]) OR ("St Martin"[Mesh]) OR ("Turks and Caicos Island"[Mesh]) OR (South American women) OR (Latin American women) OR (Caribbean women)) | 35 |
| Embase (Ovid) | ((reproductive tract infections or sexually transmitted diseases or genital tract infection or sexually transmitted infection or sexually transmitted disease or vaginal infection) AND (South America or Latin America or Caribbean) AND (pregnant women or pregnancy)).ab,kw,ti | 1 |
| SciELO | (ti:(sexually transmitted infection) OR (reproductive tract infection) OR (genital tract infection)) OR (sexually transmitted disease)) OR (vaginal infection)) AND (ti:(pregnant women))) OR (pregnancy outcome)) | 22 |
| LILACS | (sexually transmitted infection) OR (reproductive tract infection) OR (genital tract infection) OR (sexually transmitted disease) OR (vaginal infection) AND (pregnant women) OR (pregnancy outcome) | 51 |

**S3 Table.** Questions for the critical appraisal checklist for cohort studies, cross-sectional studies and case-control studies.

| **Question numbers** | **Cohort studies** | **Cross-sectional studies** | **Case-control studies** |
| --- | --- | --- | --- |
| 1 | Were the two groups similar and recruited from the same population? | Were the criteria for inclusion in the sample clearly defined? | Were the groups comparable other than the presence of disease in cases or the absence of disease in controls? |
| 2 | Were the exposures measured similarly to assign people to both exposed and unexposed groups? | Were the study subjects and the setting described in detail? | Were cases and controls matched appropriately? |
| 3 | Was the exposure measured in a valid and reliable way? | Was the exposure measured in a valid and reliable way? | Were the same criteria used for identification of cases and controls? |
| 4 | Were confounding factors identified? | Were objective, standard criteria used for measurement of the condition? | Was exposure measured in a standard, valid and reliable way? |
| 5 | Were strategies to deal with confounding factors stated? | Were confounding factors identified? | Was exposure measured in the same way for cases and controls? |
| 6 | Were the groups/participants free of the outcome at the start of the study (or at the moment of exposure)? | Were strategies to deal with confounding factors stated? | Were confounding factors identified? |
| 7 | Were the outcomes measured in a valid and reliable way? | Were the outcomes measured in a valid and reliable way? | Were strategies to deal with confounding factors stated? |
| 8 | Was the follow up time reported and sufficient to be long enough for outcomes to occur? | Was appropriate statistical analysis used? | Were outcomes assessed in a standard, valid and reliable way for cases and controls? |
| 9 | Was follow up complete, and if not, were the reasons to lose to follow up described and explored? | Not applicable | Was the exposure period of interest long enough to be meaningful? |
| 10 | Were strategies to address incomplete follow up utilized? | Not applicable | Was appropriate statistical analysis used? |
| 11 | Was appropriate statistical analysis used? | Not applicable | Not applicable |
| 12 | Not applicable | Not applicable | Not applicable |
| 13 | Not applicable | Not applicable | Not applicable |

Provided by Joanna Briggs Institute Reviewer’s Manual^44^.

**S1 Checklist.** PRISMA checklist displaying the page numbers where the section topics are provided.

| **Section/topic** | **#** | **Checklist item** | **Reported on page #** |
| --- | --- | --- | --- |
| **Title** | | | |
| Title | 1 | Identify the report as a systematic review, meta-analysis, or both. | 1 |
| **Abstract** | | | |
| Structured summary | 2 | Provide a structured summary including, as applicable: background; objectives; data sources; study eligibility criteria, participants, and interventions; study appraisal and synthesis methods; results; limitations; conclusions and implications of key findings; systematic review registration number. | 2 |
| **Introduction** | | | |
| Rationale | 3 | Describe the rationale for the review in the context of what is already known. | 3-4, S1 Table |
| Objectives | 4 | Provide an explicit statement of questions being addressed with reference to participants, interventions, comparisons, outcomes, and study design (PICOS). | 4 |
| **Methods** | | | |
| Protocol and registration | 5 | Indicate if a review protocol exists, if and where it can be accessed (e.g., Web address), and, if available, provide registration information including registration number. | Not applicable |
| Eligibility criteria | 6 | Specify study characteristics (e.g., PICOS, length of follow-up) and report characteristics (e.g., years considered, language, publication status) used as criteria for eligibility, giving rationale. | 5 |
| Information sources | 7 | Describe all information sources (e.g., databases with dates of coverage, contact with study authors to identify additional studies) in the search and date last searched. | 5-6 |
| Search | 8 | Present full electronic search strategy for at least one database, including any limits used, such that it could be repeated. | S2 Table |
| Study selection | 9 | State the process for selecting studies (i.e., screening, eligibility, included in systematic review, and, if applicable, included in the meta-analysis). | 5-7, Fig 1, Fig 2 and S3 Table |
| Data collection process | 10 | Describe method of data extraction from reports (e.g., piloted forms, independently, in duplicate) and any processes for obtaining and confirming data from investigators. | 5-7 |
| Data items | 11 | List and define all variables for which data were sought (e.g., PICOS, funding sources) and any assumptions and simplifications made. |  |
| Risk of bias in individual studies | 12 | Describe methods used for assessing risk of bias of individual studies (including specification of whether this was done at the study or outcome level), and how this information is to be used in any data synthesis. | 6, S3 Table |
| Summary measures | 13 | State the principal summary measures (e.g., risk ratio, difference in means). | 6 |
| Synthesis of results | 14 | Describe the methods of handling data and combining results of studies, if done, including measures of consistency (e.g., I^2^) for each meta-analysis. | 6-7, Table 1 |
| Risk of bias across studies | 15 | Specify any assessment of risk of bias that may affect the cumulative evidence (e.g., publication bias, selective reporting within studies). | Not applicable |
| Additional analyses | 16 | Describe methods of additional analyses (e.g., sensitivity or subgroup analyses, meta-regression), if done, indicating which were pre-specified. | Not applicable |
| **Results** | | | |
| Study selection | 17 | Give numbers of studies screened, assessed for eligibility, and included in the review, with reasons for exclusions at each stage, ideally with a flow diagram. | 6-7, Fig 1 and Fig 2 |
| Study characteristics | 18 | For each study, present characteristics for which data were extracted (e.g., study size, PICOS, follow-up period) and provide the citations. | 6-7, Table 1 and S4 Table |
| Risk of bias within studies | 19 | Present data on risk of bias of each study and, if available, any outcome level assessment (see item 12). | 9 and S5 Table |
| Results of individual studies | 20 | For all outcomes considered (benefits or harms), present, for each study: (a) simple summary data for each intervention group (b) effect estimates and confidence intervals, ideally with a forest plot. | 7-9, Table 1 and S4 Table |
| Synthesis of results | 21 | Present results of each meta-analysis done, including confidence intervals and measures of consistency. | 7-9, Table 1 |
| Risk of bias across studies | 22 | Present results of any assessment of risk of bias across studies (see Item 15). | Not applicable |
| Additional analysis | 23 | Give results of additional analyses, if done (e.g., sensitivity or subgroup analyses, meta-regression [see Item 16]). | Not applicable |
| **Discussion** | | | |
| Summary of evidence | 24 | Summarize the main findings including the strength of evidence for each main outcome; consider their relevance to key groups (e.g., healthcare providers, users, and policy makers). | 10-14 |
| Limitations | 25 | Discuss limitations at study and outcome level (e.g., risk of bias), and at review-level (e.g., incomplete retrieval of identified research, reporting bias). | 13 |
| Conclusions | 26 | Provide a general interpretation of the results in the context of other evidence, and implications for future research. | 13-14 |
| **Funding** | | | |
| Funding | 27 | Describe sources of funding for the systematic review and other support (e.g., supply of data); role of funders for the systematic review. | Not applicable |

Summary PRISMA checklist provided by Page et al^44^.

**S4 Table.** Summary of the cohort characteristics and methodological features of the retrieved studies ordered per publication year.

| **Author, year** | **Country** | **Study design** | **Study population** | **Microorga-nism** | **Follow Up** | **Methods to detect pathogen** | **Participants** | **Maternal age** | **Gravidy/**  **parity period** | **Other infections** |
| --- | --- | --- | --- | --- | --- | --- | --- | --- | --- | --- |
| Araújo et al. 2021^52^ | Brazil | Cross-sectional study | Pregnant women | *Treponema pallidum* | VDRL through pregnancy | Blood sample and VDRL | 478 pregnant women | 24.9 years old (mean) | <37 weeks of gestation | Not specified |
| Benedetti et al. 2019^53^ | Brazil | Cross-sectional study | Pregnant women attending prenatal care | *Treponema pallidum* | Successful treatment was observed only on 2 out of the TP+ patients diagnosed during antenatal care | Blood sample and VDRL + ELISA | 661 women | 26 years old (mean) | 1^st^, 2^nd^ and 3^rd^ pregnancy stages | HIV (n=5, 0.8%) |
| Casillas-Vega et al. 2017^54^ | Mexico | Cross-sectional study | First-time women attending to obstetric visit | *Chlamydia Trachomatis* | Not mentioned | Endocervical sample, PCR-RFLP and nested PCR | 662 women | 31 years old (mean) | Not specified | Not specified |
| Cardoso et al. 2015^63^ | Brazil | Cross-sectional study | Pregnant women reporting infant or fetal deaths due to CS | *Treponema pallidum* | Not mentioned | Blood sample and VDRL | Not specified | No data | Fetal and infant death occurred from 22 gestational weeks to the 7^th^ day of life | Not specified |
| Schmidt et al. 2015^55^ | Brazil | Cross-sectional study | Parturient women who had preterm birth | *Chlamydia Trachomatis* | Not mentioned | Urine sampling, DNA extraction + PCR | 323 pregnant women | 24.6 years old (mean) | Between 22 weeks and 36 weeks and 6 days | TP, *Streptoco-ccus B* and UTI |
| De Borborema-Alfaia et al. 2013^56^ | Brazil | Cross-sectional study | Pregnant women in Manaus, Amazon department | *Chlamydia Trachomatis* | Newborns were evaluated up to 24h of life and followed up to 60 days of life | Endocervical sample, DNA extraction + PCR | 88 pregnant women | 23.5 years old (mean) | From third trimester of pregnancy (29 weeks) | Not specified |
| Hitti et al. 2010^15^ | Peru | Case-control study | Pregnant women who presented spontaneous preterm birth | *Mycoplasma Genitalium* | 48h after delivery to perform sample collection | Cervicovaginal sampling *M. genitalium*-specific research-use only NAAT assay | 661 pregnant women who suffered from preterm birth at <37 weeks and 667 controls who delivered at ≥ 37 weeks | 25 years old (mean) | 20^th^ to 36^th^ week of pregnancy | CT (8%), NG (<1%) and TV (2%) |
| Southwick et al. 2001^58^ | Bolivia | Descriptive cohort study | Pregnant women | *Treponema pallidum* | Women and infants were given penicillin when diagnosed after delivery | Blood sample, RPR test and FTA-ABS | 1428 pregnant women giving birth to live-born infants | 23 years old (median) | ≥37 weeks, postpartum | Not specified |
| Guarner et al. 2000^61^ | Bolivia | Prospective cohort study | Pregnant women | *Treponema pallidum* | Penicillin as treatment for mother and children after delivery | Blood sample, RPR and FTA-ABS | 1559 pregnant women | No data | ≥37 weeks, postpartum | Not specified |
| Behets et al. 1995^59^ | Haiti | Prospective cohort study | Multiparous pregnant women | *Treponema pallidum* | Positive women to any STI received treatment at the time of diagnosis | Blood sample RPR and MHA-TP | 996 women | 25.9 years old (mean) | 1st and 2nd term of pregnancy | 35% TV+, 12% NG+ or CT+ or both, 8% HIV+ |

CS, congenital syphilis. CT, *Chlamydia trachomatis*. ELISA, enzyme-linked immunosorbent assay. FTA-ABS, treponemal antibody absorbed test. HIV, human immunodeficiency virus. MHA-TP. Microhemaglutination assay for *Treponema Pallidum*. NAAT, nucleic acid amplification test. NG, *Neisseria gonorrhoeae*. PCR, polymerase chain reaction. RPR, rapid plasma reagin. STI, sexually transmitted infection. TP, *Treponema pallidum*. TV*, Trichomonas vaginalis*. UTI, urinary tract infection. VDLR, venereal disease research laboratory.

**S5 Table.** Results of the Joanna Briggs Institute critical appraisal checklist.

| **Reference** | **Q1** | **Q2** | **Q3** | **Q4** | **Q5** | **Q6** | **Q7** | **Q8** | **Q9** | **Q10** | **Q11** | **Q12** | **Q13** | **% yes** | **Risk*** |
| --- | --- | --- | --- | --- | --- | --- | --- | --- | --- | --- | --- | --- | --- | --- | --- |
| ***Cross-sectional study*** | | | | | | | | | | | | | | | |
| Araújo et al. 2020^52^ | 🗸 | 🗸 | ? | 🗸 | 🗸 | 🗸 | 🗸 | 🗸 | N/A | N/A | N/A | N/A | N/A | 93.7% | low |
| Benedetti et al. 2019^53^ | 🗸 | 🗸 | 🗸 | 🗴 | 🗸 | 🗸 | 🗸 | 🗸 | N/A | N/A | N/A | N/A | N/A | 87.5% | low |
| Casillas-Vega et al. 2017^54^ | 🗸 | 🗸 | 🗸 | 🗴 | 🗸 | 🗴 | 🗸 | 🗸 | N/A | N/A | N/A | N/A | N/A | 75% | low |
| Cardoso et al. 2015^63^ | 🗸 | 🗸 | 🗸 | 🗸 | 🗸 | 🗴 | 🗸 | 🗸 | N/A | N/A | N/A | N/A | N/A | 87.5% | low |
| Schmidt et al. 2015^55^ | 🗸 | 🗸 | 🗸 | 🗸 | 🗸 | 🗸 | 🗸 | 🗸 | N/A | N/A | N/A | N/A | N/A | 100% | low |
| de Borborema-Alfaia et al. 2013^56^ | 🗸 | ? | 🗸 | 🗸 | 🗴 | 🗴 | 🗸 | 🗸 | N/A | N/A | N/A | N/A | N/A | 68.8% | moderate |
| ***Cohort study*** | | | | | | | | | | | | | | | |
| Southwick et al. 2001^58^ | 🗸 | 🗸 | 🗸 | 🗸 | 🗴 | 🗴 | 🗸 | 🗸 | 🗸 | N/A | 🗸 | N/A | N/A | 80% | low |
| Guarner et al. 2000^61^ | 🗸 | 🗸 | 🗸 | 🗸 | 🗴 | 🗴 | 🗸 | 🗸 | 🗸 | N/A | 🗸 | N/A | N/A | 80% | low |
| Behets et al. 1995^59^ | 🗸 | 🗸 | 🗸 | 🗸 | 🗴 | 🗴 | 🗴 | 🗸 | ? | N/A | 🗸 | N/A | N/A | 65% | moderate |
| ***Case-control study*** | | | | | | | | | | | | | | | |
| Hitti et al. 2010^15^ | 🗸 | 🗸 | 🗸 | 🗸 | 🗸 | 🗸 | 🗸 | 🗸 | 🗸 | 🗸 | N/A | N/A | N/A | 100% | low |

Qn: questions based on the JBL risk assessment (S3 Table). ✓: Indicates “yes” (1 point). 🗴: Indicates “No” (0 points). ‘?’: Indicates “unclear” (0,5 points). Risk^*^: The risk of bias was considered high when the study score ≤ 49%, moderate when the study score reached 50 to 69%, and low when the study score reached ≥ 70%. N/A = not applicable.
